# Effects of Tantalum on Microstructure Evolution and Mechanical Properties of High-Nb TiAl Alloys Reinforced by Ti_2_AlC

**DOI:** 10.34133/2019/5143179

**Published:** 2019-09-15

**Authors:** Hongze Fang, Ruirun Chen, Yong Yang, Yanqing Su, Hongsheng Ding, Jingjie Guo

**Affiliations:** ^1^National Key Laboratory for Precision Hot Processing of Metals, Harbin Institute of Technology, 150001, China; ^2^State Key Laboratory of Advanced Welding and Joining, Harbin Institute of Technology, 150001, China

## Abstract

Experiments have been carried out to study the relationship between the addition of tantalum and microstructure, especially the formation of the B2 phase in lamellar colonies. The mechanical properties, with different contents of Ta, were also measured. Ti46Al8Nb2.6C*x*Ta alloys were prepared by casting with the content of Ta varying from zero to 1.0 at.%. Experimental results show that the B2 phase forms in lamellar colonies with the addition of Ta, and its content increases when the content of Ta increases. Meanwhile, the *γ* phase decreases and the lattice parameter of the *α*_2_ phase increases. The size of the lamellar colony decreased from 29.9 to 21.6 *μ*m. Ta dissolves into Ti_2_AlC by substitution, and its solubility is more than 1.1% tested by EDS. Nb, which is necessary for the formation of the B2 phase, comes from two aspects. The first is that Ta dissolves into the Ti_2_AlC and partly replaces the Nb atom and the second is the decrease in the *γ* phase because it has higher solid solubility for Nb. The increase in Nb in the liquid phase increases the composition supercooling and heteronucleation at the solidification front, which accounts for refining the lamellar colony. Room temperature compressive testing showed that the compressive strength and the strain increased when the Ta content increased up to 0.8% and then decreased. Improvement of the compressive properties resulted from the grain boundary strengthening and their decrease induced by more content of the B2 phase. Tensile properties, at elevated temperature, were improved with testing temperature increasing from 750 to 950°C, because solid solution strengthening is a major influence factor.

## 1. Introduction

Based on previous research [1], the TiAlNb alloy had high B2 content which decreased the mechanical properties. When Ta is added into the alloy, there is no B2 phase in the microstructure. The mechanism is that the tantalum atoms impede the diffusion of the niobium atoms and more niobium atoms dissolve in the lamellar colony. However, further research is needed to verify the above mechanisms. For research, high-Nb TiAl-based alloys are promising structural materials to replace heavy, Ni-based superalloys, in turbine blades of aircraft engines, as well as in turbocharger wheels, for advanced automobile engines, at high-temperature applications between 600 and 900°C, due to their low density in combination with good creep resistance, high-temperature corrosion resistance, and good oxidation properties [2–4]. However, the development and application of high-Nb TiAl alloys are still severely restricted, in recent years, due to their low ductility at room temperature and high strain and insufficient strength at high temperature [5–8]. To overcome this deficiency, several methodologies have been developed, including directional solidification, powder metallurgy technology, and the introduction of a second reinforcing phase to form composites [9–12].

These methods have been used because the high content of niobium was added in the TiAl alloys, which will produce positive effects and negative effects. The addition of Nb into TiAl alloys increases the mechanical strength from room temperature up to 800°C, which is ascribed to the solution hardening of Nb and microstructural refinement [13–16]. The developed TiAl alloys with a high content of Nb had good tensile properties at room temperature and elevated temperature and antioxygenic property at 900°C, relative to TiAl alloys without Nb [17]. When the testing temperature was 850°C and cyclic stress-strain behavior was tested by cyclic softening, the stress amplitude rapidly saturated, at low strain amplitude, for TiAl alloys with high content of Nb [18]. Unfortunately, deformation incompatibility and formation of the microcracks at the interface between the B2 phase and TiAl matrix reduce the ductility [19, 20]. Formation of the B2 phase in the TiAl matrix influences the microstructure and mechanical properties.

To study the formation of the B2 phase, the alloying elements, content of Al, in situ particles, and content of the *γ*/*α*_2_ phase have been researched. Compared with titanium, tantalum and niobium atoms are the slow diffusers and are used in place of titanium atom in TiAl-based alloys. Tantalum is a slower diffuser than niobium because Ta has a bigger atomic radius and higher melting point [19, 20]. However, a high content of Ta results in an increase in density and cost, when comparing Ti46Al8Ta alloy to Ti46Al8Nb alloy [21, 22]. The content of aluminum is a significant element on the phase transformation and the phase contents, which directly affect the content of the alpha2 and gamma phases [23]. The content of the gamma phase and alpha2 phase affects the solid solubility of tantalum and niobium [22, 24]. Another method to control the microstructure and properties is the formation of some reinforced phases during the solidification, such as Ti_2_AlC, Ti_2_AlN, and TiB. And casting is a low-cost and convenient method. If the carbon content exceeds a certain limit in the lamellar colony, Ti_2_AlC particles precipitate. The carbon is not only an effective precipitation strengthener but also a valid solid solution strengthener [25]. The density and thermal expansion coefficient of Ti_2_AlC particles are 4.11 g/cm^3^ and 8.8 × 10^−6^ K^−1^ and are close to those of TiAl, whose corresponding values are 3.84 g/cm^3^ and 12 × 10^−6^ K^−1^ [26], which improves the preparation of the Ti_2_AlC-reinforced TiAl-based alloys by avoiding the phenomena of segregation of the ceramic particles and reducing the inner stress between the ceramic and the matrix. The ceramic phase of Ti_2_AlC usually combines properties of both metals and ceramics [27], which possess both ductility and stiffness. However, the distribution of carbides must be uniform and the length-diameter ratio of carbides needs to improve.

This research is concerned with the above scenarios. The Ti46Al8Nb2.6C-*x*Ta alloys have been designed, with low Ta content, by casting, especially the influencing mechanism of Ta that the tantalum atoms impede the diffusion of the niobium atoms and more niobium atoms dissolve in the lamellar colony. Phase composition, microstructural evolution, mechanical properties at room and elevated temperature, morphology of fracture, and related mechanisms have been investigated in detail.

## 2. Materials and Methods

Titanium sponge (purity of 99.9%), high-purity aluminum (purity of 99.9%), graphite (purity of 99.9%), tantalum powder (purity of 99.9%), and a master alloy of Al-70 wt.% Nb were used in this research. Ti46Al8Nb2.6C-*x*Ta ingots were cast in a high-vacuum arc melting furnace with the content of Ta varying from zero to 1.0 at.%. The ingots were melted five times to ensure a homogeneous composition. The weight of each ingot was about 85 g. The sizes of the ingot were 50 mm in diameter and 12 mm in height.

The chemical composition of Ti46Al8Nb2.6C alloys with different contents of Ta has been measured by the Axios-PW4400 X-ray Spectrometer to confirm the composition uniformity, and the results are shown in Table 1. Phase constituents of the ingot samples were checked by X-ray diffraction (XRD). A half ingot was obtained by wire-electrode cutting along the axis direction, after which the cut surface was polished. The buffed surface was etched for 10 seconds by a solution of 10% HF+10% HNO_3_+80% H_2_O (vol.%). Microstructure of the samples was examined by scanning electron microscopy (SEM, equipment types of Quanta 200F and Japan TM3030), and composition analyses were tested by energy-dispersive spectroscopy (EDS).

Sizes of compressive test specimens were 6 mm in diameter and 9 mm in height, which were used for compressive testing conducted on an electronic universal material testing machine (AG-X plus, 250 kN). The specimens for compressive testing were polished on both the upper and lower surfaces to ensure that the specimens were parallel with loading direction. The test specimens were measured at room and elevated temperature with a strain rate of 0.02/s. The strain and stress of the specimens were tested with an extensometer.

Sizes of the tensile specimens were 45 mm in total length, 15 mm in total width, and 2 mm in height. The gauge size was 15 mm in total length, 5 mm in total width, and 2 mm in height, which was tested using an electronic universal material testing machine (Instron 5569) at high temperature (750°C, 850°C, and 950°C). The tensile specimens were always in a resistance furnace and hung in the tensile clamp. The values of the compression and tensile tests are at failure. Each value was tested four times, and an average numerical value was achieved.

## 3. Results and Discussion

### 3.1. Microstructure Analyses of Ti46Al8Nb2.6C*x*Ta Alloys

**Figure 1:**
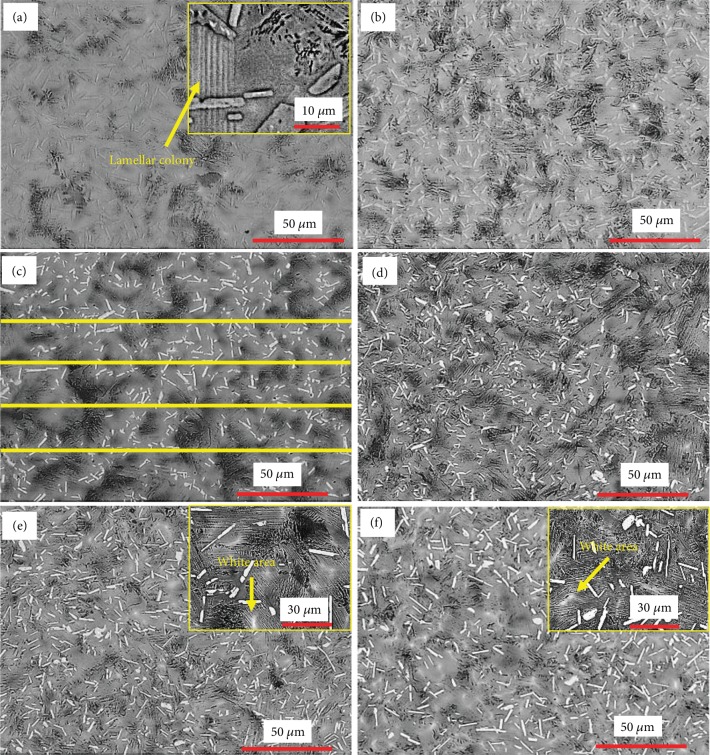
SEM images of the Ti46Al8Nb2.6C*x*Ta alloys: (a) zero; (b) 0.2 at.%; (c) 0.4 at.%; (d) 0.6 at.%; (e) 0.8 at.%; (f) 1.0 at.%.

SEM images of Ti46Al8Nb2.6C*x*Ta alloys are shown in Figure 1. It can be seen that there is no obvious white area in the initial matrix of Ti46Al8Nb2.6C alloy (Figure 1(a)). When the content of Ta increases from zero to 1.0%, the size of the lamellar colony decreases which can be seen in Figures 1(b)–1(f).

The white area obviously forms in a lamellar colony when the contents of Ta are 0.8% and 1.0%. The Ti_2_AlC phase becomes bright and there are some black regions between lamellar colonies in the Ti46Al8Nb2.6C*x*Ta alloys. To accurately research the effect of Ta on the microstructure, the lamellar colony sizes were measured by using a linear intercept method (such as Figure 1(c)), and the average results, from four tests for each sample, are shown in Table 2. The measured results in Table 2 show that the size of the lamellar colony decreases from 29.9 to 21.6 *μ*m when the content of Ta increases from zero to 1.0%.

**Figure 2:**
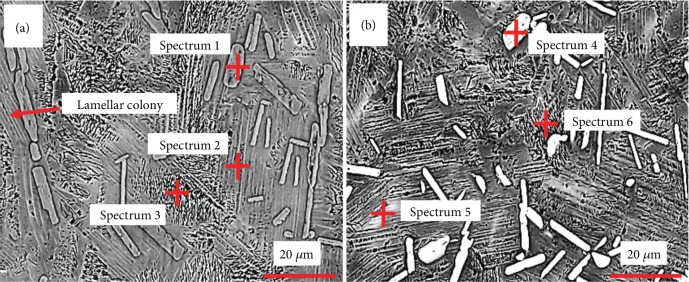
Point analysis (SEM-EDS) and high-magnification images: (a) the Ti46Al8Nb2.6C alloy; (b) the Ti46Al8Nb2.6C1.0Ta alloy.

Figure 2 shows the high-resolution SEM images and spectrum analysis of the Ti46Al8Nb2.6C and Ti46Al8Nb2.6C1.0Ta alloys. The results of the point analyses are shown in Table 3. Component analyses of spectrum 1 and spectrum 4 indicate the atomic ratios of Ti, C, and Al to be about 2 : 1 : 1 which are the values of the Ti_2_AlC phase. The black regions between the lamellar colonies have a high content of Al as seen from the results of spectrum 3 and spectrum 6. The Ti46Al8Nb2.6C alloy has a high content of Nb. The content of Nb is 8.29% from spectrum 2 in Figure 2(a), which is higher than the content of Nb in spectrum 1 and spectrum 3. There is no B2 phase in the Ti46Al8Nb2.6C alloy because more Nb dissolves into the lamellar colony and the Ti_2_AlC phase, which is the reason why the studied alloys have a big capacity to solid solubilize the niobium and have no redundant niobium to form the B2 phase in the matrix. With the addition of 1.0 at.% Ta, the white area appears in the lamellar colony. Comparing the EDS results of spectrum 5 with the EDS results of spectrum 4 and spectrum 6, the content of Nb is 10.21 at.% because the content of Nb is beyond the solid solubility of the lamellar colony and the white area forms with more content of Nb. The content of Ta in the Ti_2_AlC phase is higher than that in the matrix. In Figure 2(b), the feature of the white area forming in the lamellar colonies is the *α*-segregation [28]. The results further verify the mechanism, of the disappearance of the white area with the addition of Ta, in that the addition of Ta promotes the dissolution of Nb in the lamellar colony at a high solidification rate. Nb, as an alloying element, shows an exceptionally high solubility, up to about 10%, in the lamellar colony, which is similar to the results of the previous research [25].

### 3.2. Phase Composition of Ti46Al8Nb2.6C*x*Ta Alloys

**Figure 3:**
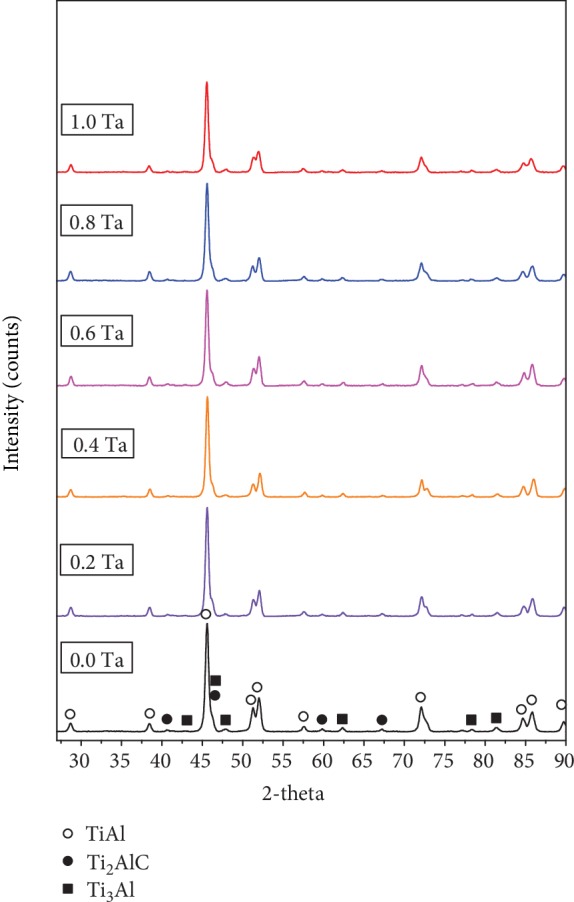
Results of XRD in the Ti46Al8Nb2.6C*x*Ta alloys.

Figure 3 shows the phase composition of the Ti46Al8Nb2.6C*x*Ta alloys by XRD. There are mainly the *γ*, *α*_2_, and Ti_2_AlC phases, which is similar to the results [29]. The peak of the *γ* phase decreases, and the peak of the *α*_2_ phase remains stable with the increase in Ta as seen in Figure 3. There are no obvious peaks of the B2 phase. The influence of Ta on the phase contents of Ti46Al8Nb2.6C*x*Ta alloys has been calculated, and the results are shown in Table 4. According to previous research [30], the content of the *ω* phase (wt.%) is defined as

(1) $$\begin{array}{c} \omega =kI, \end{array}$$

where *k* is a constant and *I* is the integral of the strongest diffraction peak. Relative contents of the phases have been calculated as

(2) $$\begin{array}{c} R_{\alpha_{2}/\gamma }=\frac{\omega_{\alpha_{2}}}{\omega_{\gamma }}=\frac{k_{\alpha_{2}}I_{\alpha_{2}}}{k_{\gamma }I_{\gamma }}=k_{\alpha_{2}/\gamma }\frac{I_{\alpha_{2}}}{I_{\gamma }},\\ R_{{\text{Ti}}_{2}\text{AlC}/\gamma }=\frac{\omega_{{\text{Ti}}_{2}\text{AlC}}}{\omega_{\gamma }}=\frac{k_{{\text{Ti}}_{2}\text{AlC}}I_{{\text{Ti}}_{2}\text{AlC}}}{k_{\gamma }I_{\gamma }}=k_{{\text{Ti}}_{2}\text{AlC}/\gamma }\frac{I_{{\text{Ti}}_{2}\text{AlC}}}{I_{\gamma }}, \end{array}$$

where *k*_*α*_2_/*γ*_ and *k*_Ti_2_AlC/*γ*_ are the relative coefficients.

**Figure 4:**
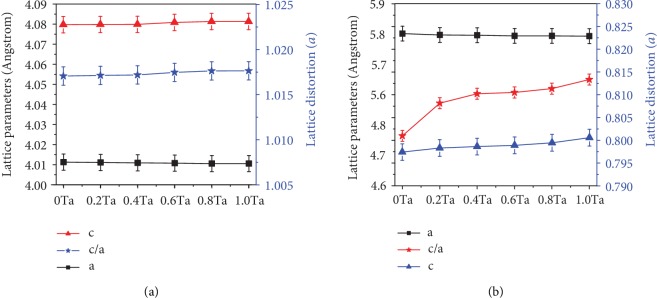
Calculating results of *a*, *c*, and *c*/*a* of Ti46Al8Nb2.6C*x*Ta alloys: (a) the gamma phase; (b) the alpha2 phase.

According to Table 4, the relative content of the Ti_2_AlC phase remains stable in the alloys. The relative content of the *γ* phase decreases from 88.6% to 83.1%, and the relative content of the *α*_2_ phase increases from 4.8% to 10.4%.  Figure 4 indicates the effects, on lattice distortion and lattice parameters, of the gamma and alpha2 phases of the Ti46Al8Nb2.6C*x*Ta alloys. The change law of lattice distortion and lattice parameters of gamma and alpha2 phases is different in Figures 4(a) and 4(b). Values of *a* decrease and those of *c* increase, at a small range, in the *γ* phase when the content of Ta increases, which increases the value of *c*/*a* as seen in Figure 4(a). Values of *a* have no change, and the value of *c* increases in the alpha2 phase when the content of Ta increases; hence, the value of *c*/*a* increases, as seen in Figure 4(b). The above results show that more alloying elements (Nb or Ta) dissolve into the *α*_2_ phase of Ti46Al8Nb2.6C*x*Ta alloys.

### 3.3. Fracture Morphology and Mechanical Property of Ti46Al8Nb2.6C*x*Ta Alloys

**Figure 5:**
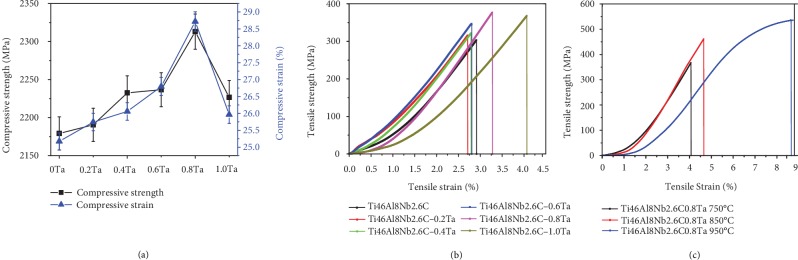
Mechanical properties of the Ti46Al8Nb2.6C*x*Ta alloys: (a) compressive properties; (b) tensile properties at 750°C; (c) tensile properties at 750°C, 850°C, and 950°C.

Mechanical properties of the Ti46Al8Nb2.6C*x*Ta alloys at room and elevated temperature are shown in Figure 5. Compressive tests show that the strength increases from 2179 to 2313 MPa, and the strain increases from 25 to 29%, when the content of Ta increases from 0 to 0.8%, as seen in Figure 5(a). The compressive properties decrease with further increases in the Ta content because there is more B2 phase forming in the lamellar colony. Tensile test results of the Ti46Al8Nb2.6C*x*Ta alloys, with a testing temperature of 750°C, is shown in Figure 5(b). When the content of Ta increases from zero to 1.0%, the strength increases from 303 to 368 MPa with the strain remaining in a range between 2.7 and 4.1%. Figure 5(c) shows the properties of Ti46Al8Nb2.6C0.8Ta alloys with testing temperatures of 750, 850, and 950°C. When the testing temperature is increased from 750 to 950°C, the strength increases from 368 to 536 MPa and the strain increases from 4.1 to 8.8%. Hence, the addition of Ta improves the mechanical property, with the alloys having excellent tensile properties at elevated temperature.

**Figure 6:**
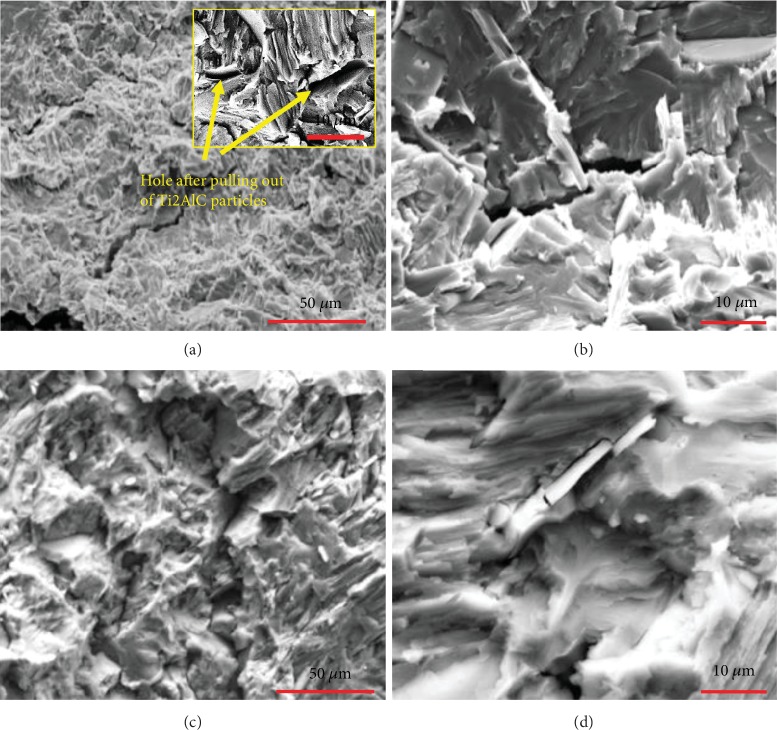
Fracture mechanism and morphology of the Ti46Al8Nb2.6C-0.8Ta alloys.

Fracture morphology of the Ti46Al8Nb2.6C0.8Ta alloys is shown in Figure 6. It can be seen that the fracture morphology is brittle fracture with cleavage surfaces and a river pattern observed by translamellar fracture and interlamellar fracture, as seen in Figure 6. The Ti_2_AlC particles act as the reinforcing phases. Particles pulled out of Ti_2_AlC particles are shown in Figures 6(b) and 6(d). A strengthening mechanism of Ti_2_AlC has the load transfer strengthening between reinforced particles and the lamellar colony, which conforms to the shear-lag model [31, 32].

## 4. Discussion

**Figure 7:**
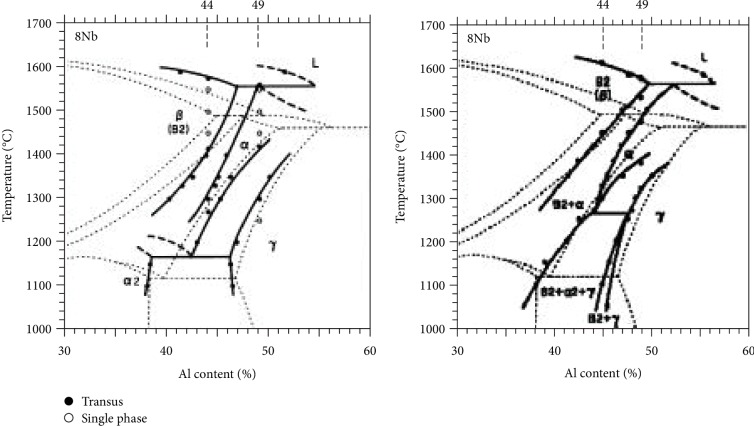
Nb-containing Ti-Al quasiphase diagrams [33]: (a) 8% Nb; (b) 10% Nb.

According to the analyses of the microstructure and phase composition, the addition of Ta is beneficial for the adjustment and control of the microstructure. The related influencing mechanisms have been discussed. There is one typical segregation of the B2 phase, which is the *α*-segregation, in Ti46Al8Nb2.6C alloys with an additional content of 0.8 and 1.0% Ta as seen in Figures 1(e) and 1(f). As the *β* phase transformation to the *α* phase is continuously taking place, the addition of Nb moves near the boundary of the alpha phase in the residual beta phase. The result of this process is that the boundaries of the alpha grains have more content of niobium. The EDS analyses indicate that the composition of the white segregation area, in the lamellar colony, is rich in Nb with a rather higher content of Al in the alloys. When the Nb content reaches 10%, as shown in Figure 7, both the phase fields of *β* + *α* + *γ* and the phase fields of *γ* + *β* appear at low temperature. When the content of Nb in the segregation area inside the *α* phase exceeds 10%, the *α* phase field transformation to the *α*_2_ + *γ* + *β* phase fields occurs, during the solidification process, leading to the formation of local lamellar structure in the *γ* + *α*_2_ + *β* phase fields. Ti46Al8Nb2.6C alloys with the addition of 0.8 and 1.0% Ta provide the condition for growing *α* phases and forming the lamellar colony with the B2 phase.

**Figure 8:**
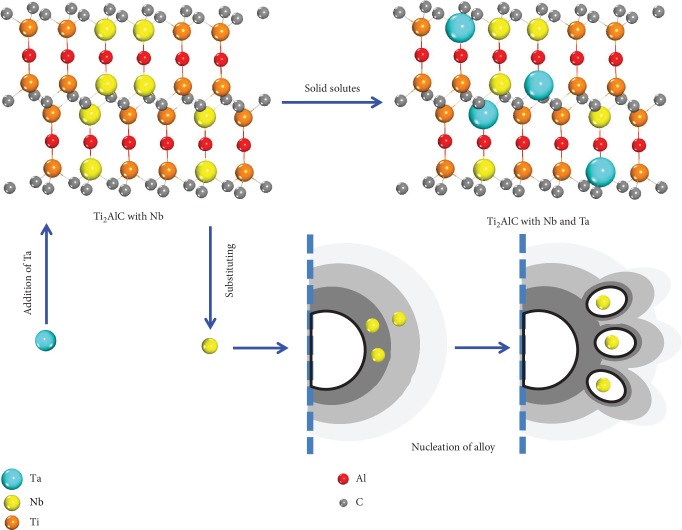
Schematic diagram of effects of Ta on the Ti_2_AlC phase.

During the solidification process at a high cooling rate, the Ta atoms impede the diffusion of Nb atoms and the lamellar colony has more content of niobium because the diffusion rate of tantalum is slower than that of niobium due to the bigger atomic radius and higher melting point of tantalum. Previous research has shown that the B2 phase disappears and the content of niobium in the lamellar colony is high with the addition of Ta [1]. The white area exists in the lamellar colony when tantalum is added into Ti46Al8Nb2.6C*x*Ta alloys because the content of Nb in the lamellar colony exceeds the solid solubility limit of the lamellar colony for Nb. There are two sources for this Nb. One source of Nb is when the Ti_2_AlC particles dissolve the certain content of Ta, and it substitutes part of the Nb, which is based on the results of Figures 1 and 2 and Table 3 and is shown in Figure 8. Another source of Nb is the decreasing content of the gamma phase when tantalum is added into the alloys. The gamma phase has an exceptionally higher solubility for Nb than the alpha2 phases, according to the previous research [34]. This research showed that the partition coefficient of Nb is 0.9 and the partition coefficient of Ta is 1.3, between the *α*_2_ phase and the *γ* phase. Hence, the gamma phase has more content of niobium and the alpha2 has more content of tantalum. The XRD results in this study have shown that the lattice parameters of the *α*_2_ phase change after the content of Nb reaches the limit of the *γ* phase (Figure 4). The content of *α*_2_ phase increases in Ti46Al8Nb2.6C*x*Ta alloys, because the *α*-segregation type of B2 phase forms, which around the B2 phase has a lower content of Al. The matrix contains a higher content of Nb and a higher content of Al to form more *α*_2_ phase.

Following classical solidification theory, constitutional undercooling results in the formation of the *β* phase with Nb ahead of the preexisting solid. Because the Nb atom is a *β*-stable element and the content of the Nb in the liquid is high enough, the formation of a new crystal nucleus has no orientation relationship to the first formed phase, as shown in Figure 8; therefore, refinement of the lamellar colony is observed. As more content of Nb from the two sources moves to the head of solidification and the content of Nb acting as the heterogeneous nucleated particles increases, the size of the lamellar colony is decreased with an increasing content of Ta. Addition of Ta elements has the effect of constitutional undercooling and heterogeneous nucleation with increasing content of Ta; the size of the lamellar colony decreases in Ti46Al8Nb2.6C*x*Ta alloys. According to previous researches [35–38], adding strongly *β*-stabilizing elements to binary TiAl alloys have revealed that the B2 phase is formed in the alloys with a fine grain microstructure. The B2 phase served as a grain refiner in TiAl-based alloys. Qiu et al. [39] reported that Ti-Al-Fe-Mo alloy had the refined lamellar colony because of adding Fe and Mo to form the B2 phase.

**Figure 9:**
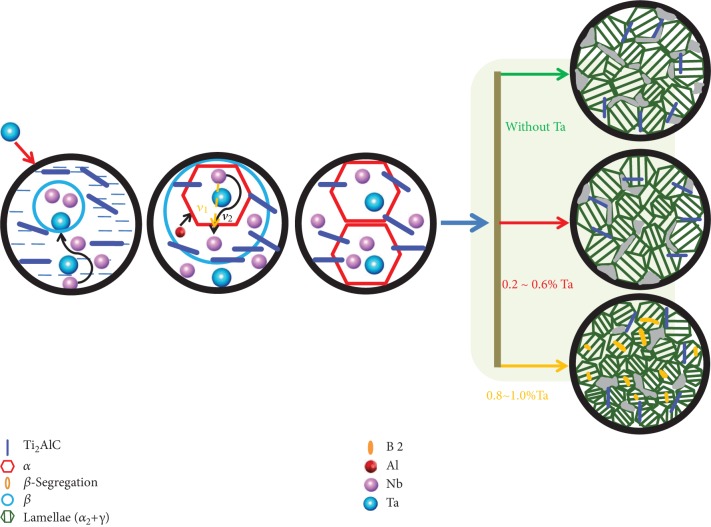
Schematic diagram of the Ti46Al2.6C8Nb*x*Ta alloys.

When Ta is added into the Ti46Al8Nb2.6C alloy, the mechanical properties are improved due to several reasons, as shown by the schematic diagram in Figure 9. Firstly, Nb and Ta have a certain degree of solid solubility in the lamellar colony and reinforced phases, according to the results from Table 3 and Figures 2 and 4. The solid solution hardening of niobium and tantalum is beneficial to the mechanical properties of Ti46Al8Nb2.6C*x*Ta alloys. Secondly, the hardening of the refined lamellar colony plays a role in controlling the microstructure and mechanical property, which is shown in Table 2. Thirdly, B2 phases are usually the source of cracks during deformation testing. The different deforming characteristics lead to microcracks at the interfaces between the B2 phase and the lamellar colony thus reducing the plasticity. Hence, the content of the B2 phase decreases and the mechanical properties increase. Finally, the composition of the phases and their content are significant factors on the influence of the mechanical properties. Plastic deformation occurs inside the gamma phases. The alpha2 phase has a structure of hcp, which has a limited slip system. Therefore, the main mechanisms for improving mechanical properties are the solid solution hardening and refined lamellar colony hardening, especially at elevated temperature. Improving tensile strength relates to reinforced particles of Ti_2_AlC containing Nb and Ta elements at high temperature. Grain boundaries usually have an irregular arrangement of atoms and several kinds of crystal imperfections. Pileup of dislocations interacts easily with crystal imperfections and disappears. Reinforcing particles hinder the movement of the imperfections during the deforming process, which has the ability to store imperfections to improve the mechanical properties. As the testing temperature increases, the effects of the solid solution hardening are greater than those of the refined lamellar colony hardening. The strain relates to the refined grain hardening. Therefore, Ti46Al8Nb2.6C*x*Ta alloys have a lower strain and higher strength. When testing temperature increases from 750 to 950°C, there is a phase decomposition process of alpha2 transforming to gamma, which happens during tertiary creep and accompanies the diffusion of aluminum. Previous researches [40, 41] have shown that this process occurs within a range of temperature from 760 to 800°C. The extent of alpha2 lamellar degeneration shows that coordination deformation, between alpha2 and gamma lamellae, promotes stress relaxation of the interfaces between them. Hence, tensile strength and strain increase with increasing temperature because of the combined action of the solid solution hardening of niobium and tantalum, precipitation strengthening by Ti_2_AlC particles, and the increasing content of gamma.

## 5. Conclusions

Ti46Al8Nb2.6C*x*Ta alloys have been prepared by arc vacuum melting. The effects of Ta on the size of the lamellar colony, phase composition, and room temperature and elevated temperature mechanical properties have been investigated. The microstructure formation and strengthening mechanism have been revealed and discussed. The following conclusions have been attained.

1. Adding Ta refines the size of the lamellar colony and its size decreases with increasing content of Ta. Refinement is due to Nb and Ta acting as heterogeneous nucleation particles
2. The content of the gamma phase decreases and the lattice parameter of the alpha2 phase increases. The B2 phase exists in the lamellar colony with the addition of Ta, and its content increases with the increasing content of Ta. More content of Ta dissolves into the Ti_2_AlC phase than the other phases
3. The necessity of Nb for the formation of the B2 phase arises from two aspects. The first is that Ta atoms dissolve into Ti_2_AlC and partly replace the Nb atoms. The second is the decrease in the *γ* phase that has a higher solid solubility for Nb
4. Room temperature tests of compression show that compressive strength and strain improve with an increasing content of Ta, up to 0.8%, and then decrease. A refined lamellar colony causes an increase in the compressive properties, but an increase in the B2 phase causes a decrease
5. Tensile strength and strain improve when testing temperature increases from 750 to 950°C. This may be due to the combined action of the solid solution hardening by niobium and tantalum, precipitation strengthening by Ti_2_AlC particles, and the increasing content of gamma

## Figures and Tables

**Table 1:** Measured chemical composition of the studied alloys.

Alloys	Ti	Al	Nb	C	Ta
Ti46Al8Nb2.6C	44.06 ± 0.88	45.45 ± 0.91	7.92 ± 0.16	2.57 ± 0.05	—
Ti46Al8Nb2.6C-0.2Ta	43.61 ± 0.87	45.76 ± 0.92	7.89 ± 0.16	2.56 ± 0.05	0.18 ± 0.01
Ti46Al8Nb2.6C-0.4Ta	43.34 ± 0.87	45.73 ± 0.91	7.96 ± 0.16	2.58 ± 0.05	0.39 ± 0.01
Ti46Al8Nb2.6C-0.6Ta	43.11 ± 0.86	45.83 ± 0.92	7.91 ± 0.16	2.59 ± 0.05	0.56 ± 0.01
Ti46Al8Nb2.6C-0.8Ta	43.08 ± 0.86	45.79 ± 0.92	7.83 ± 0.16	2.57 ± 0.05	0.73 ± 0.01
Ti46Al8Nb2.6C-1.0Ta	42.86 ± 0.86	45.74 ± 0.91	7.87 ± 0.16	2.56 ± 0.05	0.97 ± 0.02

**Table 2:** Statistical results of lamellar colony size in Ti46Al8Nb2.6C*x*Ta alloys.

Alloys	Content of Ta (at.%)	Lamellar colony size (*μ*m)
Ti46Al8Nb2.6C	0	29.9 ± 0.60
	0.2	27.8 ± 0.56
	0.4	26.9 ± 0.54
	0.6	23.4 ± 0.47
	0.8	22.4 ± 0.45
	1.0	21.6 ± 0.43

**Table 3:** Point analysis of the Ti46Al8Nb2.6C*x*Ta alloys by XRD (at.%).

Element	Ti46Al8Nb2.6C			Ti46Al8Nb2.6C1.0Ta		
	Spectrum 1	Spectrum 2	Spectrum 3	Spectrum 4	Spectrum 5	Spectrum 6
Ti	42.15	49.74	43.85	43.06	45.98	44.56
Al	29.86	41.97	48.41	26.48	43.42	48.50
Nb	6.87	8.29	7.74	5.68	10.21	6.58
C	21.12	—	—	23.24	—	—
Ta	—	—	—	1.54	0.39	0.36

**Table 4:** Relative contents of phases with different Ta contents determined by XRD (%).

Alloys	Content of Ta (%)	Benchmark phase of TiAl (%)	Relative content of Ti_3_Al ( *k*_*α*_2_/*γ*_, %)	Relative content of Ti_2_AlC (*k*_Ti_2_AlC/*γ*_, %)
Ti46Al8Nb2.6C*x*Ta	0	88.6	4.8	6.6
	0.2	87.4	5.9	6.5
	0.4	86.5	6.8	6.7
	0.6	84.9	8.3	6.1
	0.8	83.8	9.8	6.4
	1.0	83.1	10.4	6.5
